# A Clinician Perspective for a Personalized Approach to Management of Chronic Immune Thrombocytopenia with Targeted Therapies Alone or in Combination

**DOI:** 10.3390/jcm15041625

**Published:** 2026-02-20

**Authors:** María-Eva Mingot-Castellano, Michele P. Lambert, Elizabeth Bowhay-Carnes

**Affiliations:** 1 Hospital Universitario Virgen del Rocio, Instituto de Biomedicina de Sevilla (IBIS/CSIC), Universidad de Sevilla, 41013 Sevilla, Spain; 2 Department of Pediatrics, Perelman School of Medicine, University of Pennsylvania, Philadelphia, PA 19104, USA; 3 Division of Hematology, Children’s Hospital of Philadelphia (CHOP), Philadelphia, PA 19104, USA; 4 Division of Hematology-Oncology, Mays Cancer Center at UT Health MD Anderson, San Antonio, TX 78229, USA; bowhay@uthscsa.edu

**Keywords:** immune thrombocytopenia, fostamatinib, thrombopoietin receptor agonists, clinical management

## Abstract

Key mechanisms underlying immune thrombocytopenia (ITP) pathophysiology include impaired platelet production and macrophage-mediated platelet destruction, the latter of which is the disease driver in more than half of patients. Traditional sequential treatment approaches achieve suboptimal responses in many patients. This review summarizes ITP pathogenesis and the treatment landscape and proposes a personalized treatment approach for ITP after first-line treatment (corticosteroids, intravenous immunoglobulin, anti-D therapy) based on targeting underlying disease mechanisms with immunomodulatory and bone marrow-supportive therapies (fostamatinib, rituximab, and thrombopoietin receptor agonists [TPO-RAs]) prior to proceeding to later-line therapies. Clinical evidence of monotherapy and real-world studies of combination therapy are reviewed to support mechanism-based treatment selection, focusing on the complementary actions of fostamatinib (to target platelet destruction) and TPO-RAs (to stimulate platelet production). In prior studies, fostamatinib with or without TPO-RAs demonstrated durable platelet responses and manageable safety as second-line or later ITP treatment. The proposed treatment framework augments guidelines by recommending fostamatinib, rituximab, or TPO-RAs as second-line therapy options based on patient-specific disease characteristics and risks. Patients with inadequate response to fostamatinib or TPO-RA monotherapy may combine these therapies to address both platelet destruction and platelet production deficits. This novel framework tailors therapy to patient-specific pathophysiology by preferentially targeting both impaired platelet production and increased platelet destruction to support individualized care.

## 1. Introduction

Immune thrombocytopenia (ITP) is an acquired autoimmune disease characterized by a decreased platelet count (<100 × 10^9^/L) that leads to increased bruising and variable bleeding [1,2,3]. ITP has an incidence of ~2 to 4 per 100,000 people per year, affecting both adults and children, and it is typically more common in adult women than men prior to age 70 years [1,4,5]. Among newly diagnosed patients, 80% have primary ITP (characterized by isolated autoimmune thrombocytopenia), and 60–70% develop persistent or chronic ITP [3,6]. Secondary ITP is triggered by or associated with another disease or condition or by certain drugs; it may respond to treatment of the underlying disease [6,7].

The major goals of ITP treatment are to ensure a safe platelet count, minimize significant bleeding, and optimize quality of life [1,8,9]. Although 60–80% of patients respond to established therapies, over 50% of patients fail to maintain a sustained response, and many exhibit consistently low platelet counts in spite of treatment [9,10]. There continues to be a need for long-term effective and tolerable treatment approaches, and clinicians may benefit from expanded knowledge of treatments and how to use them most effectively in clinical practice [9,11].

The objective of this review is to share a unique approach to personalize ITP treatment by preferentially targeting underlying disease mechanisms using immunomodulatory and bone marrow-supportive therapies (fostamatinib and thrombopoietin receptor agonists [TPO-RAs]), consistent with the authors’ own clinical practice. This approach is not meant to act as a guideline or comprehensive review of available treatments, but rather as a clinician’s perspective on how to utilize treatment and disease mechanisms to individualize patient care in the setting of adult chronic ITP. Recent treatment guidelines, FDA approvals, clinical trial reports, and other peer-reviewed studies were consulted with a focus on publications from the last decade. The strength of published data was evaluated in the context of the authors’ clinical experience to develop this treatment approach. Background on ITP pathogenesis, recommended management of ITP, and available treatments are reviewed to aid clinicians in personalizing care. The focus of this review is to clarify the roles of fostamatinib, TPO-RAs, and rituximab in the early management of ITP to address unmet needs that remain when following existing treatment guidelines. The relevance of this approach in the context of personalized medicine is supported by clinical trial and real-world evidence.

## 2. Pathogenesis of Immune Thrombocytopenia

**Figure 1:**
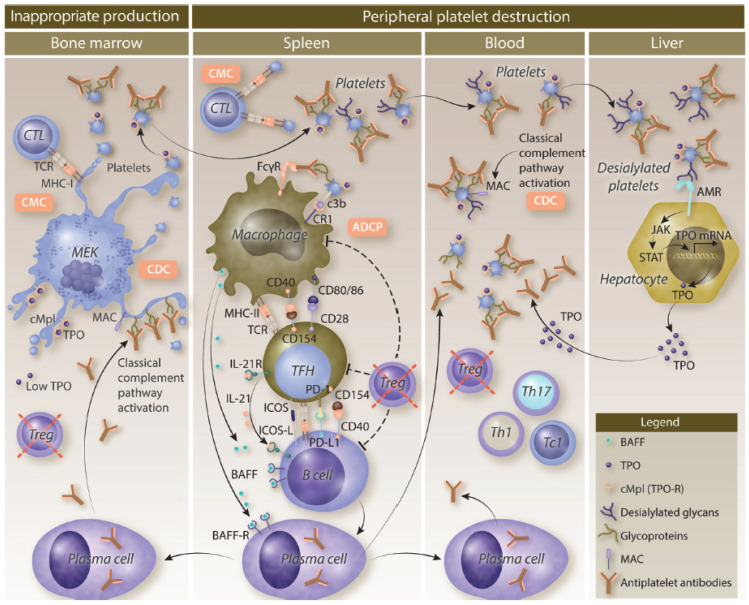
Pathogenesis of immune thrombocytopenia. ADCC, antibody-dependent cellular cytotoxicity; ADCP, antibody-dependent cellular phagocytosis; AMR, Ashwell-Morell receptor; BAFF, B-cell activating factor; CD, cluster of differentiation; CDC, complement-dependent cytotoxicity; CMC, cytotoxic T lymphocyte-mediated cytotoxicity; cMpl, thrombopoietin receptor; CR, complement receptor; CTL, cytotoxic T cell; FcγR, Fc gamma receptor; GP, glycoprotein; ICOS, inducible T-cell costimulator; ICOS-L, ICOS ligand; IL, interleukin; ITP, immune thrombocytopenia; JAK, Janus kinase; MAC, membrane attack complex; MEK, megakaryocyte; MHC, major histocompatibility complex; mRNA, messenger RNA; PD-1, programmed cell death protein 1 (CD279); PD-L1, programmed cell death ligand 1 (CD274); STAT, signal transducer and activator of transcription; Tc, cytotoxic T cell; TCR, T-cell receptor; TFH, T follicular helper cell; Th, helper T cell; TPO, thrombopoietin; Treg, regulatory T cell. Used with permission from Audia et al. [13]; permission conveyed through Copyright Clearance Center, Inc.

Primary ITP is characterized by complex mechanisms that involve both increased platelet destruction and impaired platelet production (Figure 1) [6,12,13].

Peripheral destruction of platelets is primarily driven by autoantibodies that target platelet surface glycoproteins such as GPIIb/IIIa and GPIb/IX [14,15]. These autoantibody-coated platelets are recognized and cleared by macrophages in the spleen and liver through Fcγ receptor-mediated phagocytosis [15,16,17]. In addition to antibody-mediated mechanisms, cytotoxic T lymphocytes can cause platelet lysis, further contributing to destruction [15,16]. Apoptosis of platelets, characterized by increased phosphatidylserine exposure and caspase activation, also plays a role in accelerating the platelet clearance observed in ITP [14]. This enhanced peripheral destruction is a key factor in patients with ITP.

Complement activation is another potential contributor to platelet destruction in ITP [18]. Autoantibodies fix complement on the surface of platelets, leading to enhanced clearance via complement receptors on macrophages and through direct complement-mediated cytolysis [18]. Studies have shown that plasma from patients with ITP exhibits increased complement activation capacity, which correlates with lower platelet counts and a reduced immature platelet fraction [18]. Complement fixation both promotes the removal of opsonized platelets and damages megakaryocytes, subsequently impacting platelet production [18]. The role of the complement system in ITP pathogenesis has therapeutic implications, as agents targeting complement components may offer promise as potential treatments.

Impaired platelet production is a significant factor in ITP pathophysiology. Autoantibodies target megakaryocytes, leading to dysfunction or apoptosis [14]. Morphological studies have demonstrated megakaryocyte damage and reduced platelet production in a majority of patients with ITP [14]. Additionally, T cell-mediated cytotoxicity against megakaryocytes and inhibitory cytokines further suppress thrombopoiesis [15,19]. The clinical relevance of impaired platelet production is supported by the efficacy of TPO-RAs, which stimulate megakaryocyte proliferation and platelet production in patients with ITP [15,19].

## 3. Prothrombotic Characteristics of Immune Thrombocytopenia

With an incidence twice that of population-based controls, thrombotic events such as venous and arterial thromboses affect up to 8% of patients with ITP [20,21,22,23,24,25]. In these patients, ITP itself, underlying comorbidities, or ITP therapies may be the cause of thrombosis [22]. In ITP, preactivated platelets and elevated levels of thrombin, factor VIII, and von Willebrand factor combine to generate a hypercoagulable state, putting patients at risk of thrombosis [26]. In addition, microparticles composed of small surface membrane vesicles and proinflammatory cytokines contribute to the procoagulant environment [21,23,24,27]. Increased neutrophil extracellular trap (NET) formation has been observed in patients with ITP, which can also contribute to increased thrombotic risk by providing a scaffold for platelet adhesion that promotes immunothrombosis [28,29,30]. Prothrombotic comorbidities include coronary artery disease, hypertension, diabetes, dyslipidemia, cancer, and atrial fibrillation [22,31]. The impact of associated antiphospholipid antibodies should also be considered, as 25–75% of patients with ITP are antiphospholipid antibody-positive; these antibodies further activate complement, causing an increased degree of platelet aggregation, thus enhancing the risk of thrombosis [21,27,32,33,34]. Some ITP treatments have been implicated in the elevated risk of thrombosis, such as TPO-RAs, corticosteroids, and splenectomy [22,31].

## 4. Management of Immune Thrombocytopenia

### Overview of Treatment Guidelines

Various guidelines and expert opinion reviews have been published that provide recommendations for the diagnosis and management of ITP, specifically, who to treat, the goals of treatment, and patient management by the International Consensus Report (ICR), the American Society of Hematology (ASH), the Spanish ITP Working Group (GEPTI), and others [1,5,7,35,36].

Diagnosis of ITP is one of exclusion and is based on a thorough evaluation of the patient, which should include patient and family history, physical examination, laboratory investigations (complete blood count and reticulocyte count, peripheral blood film, quantitative immunoglobulin level measurement, blood group [Rh]), and viral testing (HIV, hepatitis C virus, and hepatitis B virus) [7]. Asymptomatic patients who have mild (defined as a platelet count ≥100 to <150 × 10^9^/L) or moderate (defined as a platelet count ≥50 to <100 × 10^9^/L) thrombocytopenia should undergo observation, and patients with a higher risk of bleeding or a platelet count <30 × 10^9^/L should receive individualized treatment; however, the platelet level necessitating treatment may vary for certain patient subpopulations, such as those who are pregnant, those who receive antiplatelet or anticoagulant drugs, or those who participate in activities with a risk of severe trauma [7,35,37,38,39].

The goals of treatment are to manage bleeding, maintain a safe platelet count (e.g., ≥20–30 × 10^9^/L), improve health-related quality of life (HRQoL), and minimize treatment toxicity [1,7]. Treatment should be individualized to each patient, taking into consideration factors such as patient age, the degree of bleeding and comorbidities that may increase the risk of bleeding, concomitant medications, risk of adverse events, current quality of life, and patient expectations [7,35].

Initial treatment for newly diagnosed patients typically involves corticosteroids (prednisone, dexamethasone, methylprednisolone); if a patient has active bleeding, is at high risk for bleeding, is scheduled for surgery, is contraindicated for high-dose corticosteroids, or has not responded to corticosteroids, then intravenous immunoglobin (IVIg) or IV anti-D therapy is recommended [7]. The second-line treatments that are recommended include TPO-RAs (eltrombopag, avatrombopag, or romiplostim), rituximab, and fostamatinib [5,7,35].

Medical therapies that are suggested for subsequent treatment include immunosuppressive agents such as mycophenolate mofetil (MMF), cyclosporine A, azathioprine, danazol, and dapsone [7]. For refractory patients who have failed multiple treatments, no definitive recommendations have been made, but several options have been suggested [5,7]. Combination therapy can be considered; ideally, drugs with different mechanisms of action (MoAs) should be chosen (see Combination therapy with fostamatinib and thrombopoietin receptor agonists) [5]. Switching from one TPO-RA to another one, use of splenectomy, or hematopoietic stem cell transplantation (HSCT) in rare cases may also be considered [7].

## 5. Treatment Landscape Overview

### 5.1. First-Line Treatment Options: Corticosteroids, Intravenous Immunoglobulin, and Anti-D Therapy

The standard of care for first-line treatment is a short course (6–8 weeks maximum) of corticosteroids [7,35]. Corticosteroids exert their beneficial effects by decreasing platelet clearance and increasing production of platelets; their direct effect on blood vessels may reduce bleeding as well [7,40,41]. In addition, corticosteroids broadly act on immune cells such as macrophages, B cells, and T cells to inhibit destruction of platelets [13]. Although 60–80% of patients with ITP initially respond to the treatment, the adverse events of corticosteroids (e.g., weight gain, insomnia, acne, mood changes, risk of fracture, GI symptoms [gastric irritation or ulcer formation], and glucose intolerance) make them unsuitable for long-term use [6,35]. The fact that some patients do not respond to treatment likely reflects the heterogeneity of ITP pathophysiology described above [14]. Drug–drug interactions (DDIs) with corticosteroids can occur spanning multiple therapeutic classes, which include medications such as antibiotics, HMG-CoA reductase inhibitors, anti-inflammatories, antiretrovirals, antifungals, alkylating agents, kinase inhibitors, and anticonvulsants [42,43]. The broad interaction profile reflects the complex effects on hepatic enzyme systems by corticosteroids and the influence on drug metabolism pathways [42,43].

IVIg, another recommended first-line therapy, was approved in the U.S. in 1980 and was initially intended for treatment of primary immunodeficiency diseases [44]. IVIg reduces platelet destruction via inhibition of splenic macrophages and also enhances the clearance of antiplatelet antibodies through saturation of neonatal fragment crystallizable receptors (FcRn) [6]. The majority of patients (80%) experience a transient response lasting approximately 1–4 weeks [6]. IVIg is often used as a bridging therapy to maintain a stable platelet count prior to initiation of a more long-term treatment [45]. The most common adverse events with IVIg are associated with infusion-related reactions and include fever, chills, fatigue, nausea and vomiting, muscle and joint pain, flushing, and rash; aseptic meningitis, edema, renal failure, and thrombosis have also been reported [5,45]. DDIs may occur with concurrent administration of biologic therapies due to competitive FcRn binding, which reduces exposure to the biologic [46].

Intravenous anti-D treatment was approved by the US Food and Drug Administration (FDA) in 1995 for treatment of ITP in Rh-positive, non-splenectomized patients as an alternative first-line treatment [47]. Like IVIg, it may also be used for patients with bleeding, those who are at high risk of bleeding, those who have surgery scheduled, or those who do not respond to corticosteroids [7]. The overall response rate is 65% and lasts a median 25 days; however, adverse events reported for this therapy are serious (multisystem organ failure, acute respiratory distress, renal failure, disseminated intravascular coagulation) and warrant caution when prescribing the treatment for patients with active autoimmune hemolysis or anemia, as intravascular hemolysis and severe anemia have been reported [7,48].

### 5.2. Recommended Treatments for Second-Line and Later Settings

TPO-RAs, fostamatinib, and rituximab are recommended as second-line treatment options (Table 1) [1,5,7,35]. The TPO-RAs consist of small molecules (avatrombopag, eltrombopag) and a peptide (romiplostim) that stimulate platelet production by activating the Janus kinase-signal transducer and activator of transcription 5 (JAK/STAT5) pathway in megakaryocytes [49]. Fostamatinib prevents phagocytosis of platelets by macrophages and may also inhibit activation of antibody-generating B cells that contribute to platelet depletion via inhibition of spleen tyrosine kinase (SYK) [6]. Inhibition of SYK may reduce pathogenic Fc-receptor-driven neutrophil activation and NET formation, potentially mitigating NET-driven prothrombotic pathways [30,50,51]. Rituximab is not approved to treat ITP but is recommended for ITP in the second-line setting [5,7]. It is a monoclonal antibody that targets CD20 on B cells, triggering apoptosis of lymphocytes through two pathways: antibody-dependent cell-mediated cytotoxicity or cell lysis mediated by complement [6]. These treatments have demonstrated platelet responses in more than half of patients with ITP in second-line or later settings, and come with distinct safety considerations and drug interactions (Table 1).

Upon second-line treatment failure, recommended third-line therapies include a number of immunosuppressive agents [5,7]. Immunosuppressant drugs such as azathioprine, MMF, cyclophosphamide pulse therapy, danazol, and dapsone inhibit T and B cells, but to date, there is a lack of clinical data supporting their use for ITP treatment [6,7,52]. Response rates vary depending on the therapy used, but they have been reported to generally range from 30% to 60% (for azathioprine, MMF, danazol, and dapsone; 85% for cyclophosphamide pulse therapy), with responses achieved beginning at 1 week up to 6 months [6,9]. Adverse events of azathioprine include weakness, sweating, neutropenia, increased liver function, and increased risk of cancer; for MMF, headache, GI symptoms, fungal skin infections, and increased risk of cancer; for cyclophosphamide pulse therapy, neutropenia, infections, and deep venous thrombosis; for danazol, hirsutism, acne, amenorrhea, hepatic dysfunction; and for dapsone, GI symptoms, methemoglobinemia, rash, hemolytic anemia in those with glucose-6-phosphate dehydrogenase deficiency [6].

### 5.3. Combination Therapy with Fostamatinib and Thrombopoietin Receptor Agonists

Combining fostamatinib with other therapies is a treatment strategy that has shown promising results in real-world clinical practice (Table 2). In a single-center case series (*n* = 15), fostamatinib was combined with either prednisone, eltrombopag, or romiplostim (depending on the treatment that was ongoing at the time of fostamatinib initiation). Eighty percent of patients (12/15) achieved a response (platelet count ≥30 × 10^9^/L and at least twofold increase in the baseline count with absence of bleeding) in a median 9 days and 73% achieved a complete response (platelet count ≥100 × 10^9^/L and absence of bleeding) after a median 13 days [2,53]. During the follow-up period (median, 119 days), 40% of patients were able to discontinue steroid or TPO-RA therapy after a median 80 days. No severe adverse events were reported, and new hypertension (in 3/15 patients) was well managed with treatment. 

Another case series of patients with ITP (*n* = 5) who transitioned from TPO-RAs (which included romiplostim, eltrombopag, and avatrombopag) to fostamatinib therapy demonstrated that the combinations were effective and well tolerated, as all patients achieved platelet counts of 100 × 10^9^/L or more with no adverse events reported [54]. In an international, multicenter, retrospective study, the combination of avatrombopag with fostamatinib was effective: a response (defined as a platelet count >30 × 10^9^/L) was achieved in 7/18 patients, and 8/18 achieved a complete response (defined as a platelet count >100 × 10^9^/L), with a median time to best response of 15 days. Adverse events occurred in 33% of patients (6/18) and included headache and grade 2 liver toxicity determined to be related to avatrombopag, and grade 1 diarrhea and grade 4 neutropenia determined to be related to fostamatinib; these events were in line with known safety data [55].

TPO-RAs have also been used in combination with immunosuppressive agents or steroids. In a retrospective, observational study that included patients with multirefractory ITP, eltrombopag or romiplostim was taken with MMF, azathioprine, cyclophosphamide, cyclosporin, or everolimus [56]. Thirty out of 39 patients (77%) achieved a response, with 24 patients achieving a complete response within a median 30 days (median duration of response, 15 months); however, severe treatment-related adverse events were observed in 31% [56]. Another retrospective, observational study of patients with multirefractory ITP examined combination therapy of a TPO-RA (eltrombopag, romiplostim, or avatrombopag) with an immunosuppressive or immunomodulatory treatment (corticosteroids, purine synthesis inhibitors, or fostamatinib) [57]. Seventy-five out of 97 patients (77.3%) achieved platelet counts of ≥30 × 10^9^/L with at least doubling of baseline counts, with a median time to response of 14 days [57]. The most common safety event reported was infection requiring treatment in 15 patients (15.5%), 14 of whom required hospitalization [57]. A case series in patients with multirefractory ITP who were unresponsive to TPO-RAs showed that the addition of prednisone to their treatment regimen improved outcomes by increasing the platelet count to acceptable levels (consistently above 30 × 10^9^/L) [58]. Adverse effects were observed in 60% of patients which included headaches, hypertension, elevated liver enzymes on eltrombopag, avascular necrosis of the hip, hyperglycemia, mood changes, and weight gain [58]. In addition, an open-label study assessing eltrombopag in combination with dexamethasone showed that the combination was effective, with all patients (*n* = 12) achieving a response by day 33 and 50% having a platelet count ≥100 × 10^9^/L after 6 months [59]. These results support further investigation of combination therapies that include TPO-RAs or fostamatinib in prospective randomized clinical trials for patients with ITP.

### 5.4. Splenectomy

Splenectomy is generally only recommended for surgical candidates (few comorbidities, up-to-date immunizations) following failure of pharmacological therapies and after 1 to 2 years after ITP diagnosis [1,7,35,60]. The response to splenectomy has been reported to be sustained for a decade in 78% of patients, with relapse-free survival rates ranging from 63% to 94%; despite these encouraging statistics, almost 19% of patients have no formal response to the procedure [61,62]. The risk for postoperative complications must also be considered and includes infections, thrombosis, and hemorrhage; some events may be fatal [63].

### 5.5. New and Emerging Treatments

ITP treatment is undergoing rapid advancement, with emerging therapies designed to target specific disease mechanisms and help address the unmet needs of many patients [64]. Rilzabrutinib was recently FDA-approved for ITP, and a variety of other agents that target novel immune pathways (such as blockade of antibody reuptake mechanisms, inhibition of immune cells, and the complement cascade) are currently undergoing investigation (belimumab, ianalumab, bortezomib, sutimlimab, efgartigimod, iptacopan, daratumumab, and mezagitamab) [64]. Although evidence collected to date suggests that these latter medications may offer promise as effective therapeutic options in the future, more analysis is needed to evaluate their efficacy and safety [64].

### 5.6. Newer Management Approaches Contrasted with Traditional Guidelines

Newer management approaches for patients with ITP suggest incorporating TPO-RAs into the treatment paradigm earlier, as first-line options, which contrasts with traditional guidelines that have prioritized corticosteroids despite high relapse rates and metabolic complications [7,65,66]. Additionally, while splenectomy remains a second-line option in older protocols, more recent strategies include use of targeted biologics such as neonatal Fc receptor antagonists and complement pathway blockers, which address specific immune mechanisms rather than providing general immune suppression [66]. The 2019 ASH guidelines suggest IVIg or IV anti-D as acute interventions, but emerging therapies such as efgartigimod may offer alternatives to reduce pathogenic antibody recycling [7,67]. The traditional sequencing of therapy is currently undergoing reevaluation, challenged by promising combination therapies that target deficient platelet production and increased platelet destruction simultaneously (such as in trials that have combined TPO-RAs with immunomodulators) [53]. Recent shifts in the treatment landscape also emphasize the importance of taking into consideration HRQoL, cumulative treatment toxicity, and the cost of treatment, moving beyond just platelet count thresholds to guide treatment choices and resource utilization [65,66].

## 6. Personalized Approach for Immune Thrombocytopenia Treatment by Targeting Underlying ITP Mechanism with Fostamatinib and TPO-RAs

Published guidelines from the ICR recommend TPO-RAs, rituximab and fostamatinib as second-line treatment options, while the ASH guidelines published that same year did not include fostamatinib among recommendations for second-line therapy [7,35]. This discrepancy was due to the timing of evidence collected for appraisal (the ASH panel evaluated data published only up until May 2017, while the ICR group included articles published by July 2018) [7,35]. Analyses of fostamatinib in the second-line setting have since reported platelet responses ≥50 × 10^9^/L among 78% (25/32) of evaluated patients, with 64% (16/25) of responders demonstrating a consistent platelet response ≥50 × 10^9^/L [68]. The proposed treatment approach outlined below incorporates fostamatinib earlier in the sequencing of therapeutic agents based on real-world and clinical data demonstrating its effectiveness and manageable safety profile and is designed to offer healthcare providers a novel framework with clinical value.

**Figure 2:**
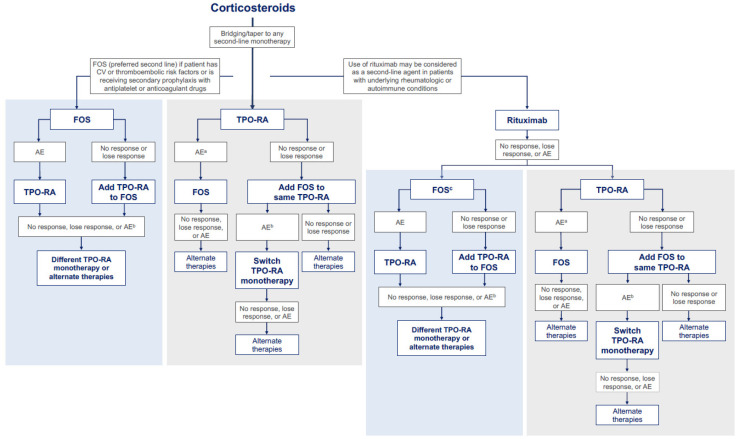
Proposed decision-making framework to personalize treatment by targeting ITP mechanisms. AE, adverse event; CS, corticosteroids; CV, cardiovascular; FOS, fostamatinib; ITP, immune thrombocytopenia; TPO-RA, thrombopoietin receptor agonist. Immunoglobulins are reserved for emergency situations requiring rapid response. If the dominant ITP mechanism is known (e.g., increased platelet destruction, impaired platelet production), this information can be incorporated into treatment decisions to personalize care. ^a^ In cases of thrombosis during TPO-RA therapy, the TPO-RA dose is reduced to maintain a safe platelet count for anticoagulation, with a gradual transition to fostamatinib over 3–4 weeks once the acute event has stabilized. ^b^ If an adverse event occurs at the current dose of fostamatinib, the dose of fostamatinib may be reduced prior to considering a switch in therapy. ^c^ Fostamatinib is preferred in patients with known platelet destruction pathophysiology, in those with CV or thromboembolic risk, or in those who are receiving antiplatelet or anticoagulant drugs.

Since ITP is a heterogeneous disease that may manifest differently in each patient, selection of the most appropriate second-line treatment option may depend on determining whether platelet destruction by macrophages or inadequate platelet production is responsible for an insufficient platelet count at the time of patient evaluation (Figure 2) [65]. A more nuanced understanding of the mechanisms underlying the patient-specific disease pathophysiology offers individual-level insight to providers and patients and can guide selection of the next appropriate therapy. This approach is designed to take advantage of the complementary mechanisms of action of fostamatinib, which inhibits platelet destruction, and TPO-RAs, which stimulate platelet production.

On the basis of clinical and real-world evidence in a small number of patients, and while taking into account the underlying drug mechanism of action, treatment with fostamatinib monotherapy as the first-choice second-line therapy may be beneficial in patients who have an inadequate response or are experiencing intolerance to corticosteroids, particularly in those who are also at high risk of cardiovascular or thromboembolic events, or who are receiving secondary prophylaxis with antiplatelet or anticoagulant drugs [5,53,55,68,69,70,71,72,73,74,75]. Fostamatinib could be added to corticosteroids that are then tapered with the intention of transitioning to fostamatinib monotherapy, which can be initiated to address platelet destruction by macrophages. If the response to fostamatinib is inadequate, a TPO-RA can be added to overcome a potential platelet production issue (Table 2). If the combination is intolerable, then reduction in fostamatinib dose can be considered, or the TPO-RA can be used alone. Patients with ITP who are intolerant of or who have an inadequate response to fostamatinib could be switched to a TPO-RA to overcome impaired platelet production. This proposed treatment sequencing requires additional evidence and should be further investigated in clinical trials and real-word practice.

Use of a TPO-RA as a single second-line agent may not be informative in determining the underlying disease mechanism leading to low platelet counts. In a patient who initiates a TPO-RA after corticosteroid therapy and experiences an inadequate response, we propose adding fostamatinib to the current TPO-RA to assess whether the increased number of platelets can be spared from destruction by macrophages [54]. If the combination is intolerable, an alternative TPO-RA monotherapy could be considered. In the case of intolerance to the initial TPO-RA, the therapy may be replaced with fostamatinib monotherapy. In order to determine if fostamatinib is efficacious, the production of platelets must increase on its own after treatment with the TPO-RA is terminated, indicating that platelet destruction has been inhibited.

Rituximab might be considered in a patient with underlying rheumatologic or autoimmune conditions. Similarly to fostamatinib and TPO-RAs, rituximab could be added to corticosteroids that are then tapered to transition to rituximab monotherapy. If a patient experiences intolerability or inadequate response to rituximab, fostamatinib or TPO-RA monotherapy could be considered as a third-line therapy. As in the second-line setting described above, fostamatinib may be beneficial in patients with known platelet destruction pathophysiology, in those with cardiovascular or thrombotic risk factors, or in those who are receiving antiplatelet or anticoagulant drugs. Subsequent treatment should follow a similar approach as in the case of second-line fostamatinib or TPO-RA monotherapy.

If these approaches fail to improve platelet count, other therapies, such as splenectomy, azathioprine, cyclosporin A, cyclophosphamide, danazol, dapsone, MMF, sirolimus/everolimus, or participation in clinical trials may be initiated.

## 7. Considerations

When first-line treatments cannot be used due to tolerability issues, there are factors that should be considered when selecting the next therapy. The benefit-to-risk ratio should be carefully examined when weighing potential efficacy against toxicity in the selection of the subsequent agent or procedure. The patient-specific platelet target should be determined, especially in those patients with thrombotic risk, as some treatments may increase this risk (such as TPO-RAs, with an incidence of 2.6–8.9% in thromboembolic events in studies lasting 2–8 years; corticosteroids, a hazard ratio of 3.3 (95% CI, 1.0–11.0) for venous thromboembolism and arterial thromboembolism with prednisone use; and splenectomy, with a prevalence of venous thromboembolism ranging from 1.4% to 16%) [60,69,76,77]. Measurement of the immature platelet fraction can provide insight into a patient’s relative risk of bleeding or thrombosis due to the reactivity of immature platelets [76]. A lower fraction of immature platelets is associated with an increased risk of bleeding and a reduced likelihood of thrombosis, whereas a higher fraction is correlated with the opposite [76]. In addition, care should be taken to ensure the use of minimally effective doses and to taper concomitant medications when indicated in order to avoid or minimize potential adverse events.

## 8. Clinical Implications

When considering the sequencing of second-line therapies for patients with ITP, particularly for those with high thrombotic risk and those with comorbidities or contraindications to other medical treatments, it may be beneficial to position fostamatinib earlier in therapy [1,5,78,79,80].

The literature suggests that managing patients with refractory ITP requires a multimodal approach integrating novel targeted therapies with strategic treatment sequencing and combination regimens. Combination therapies targeting multiple pathogenic mechanisms concurrently show improved efficacy compared to single agents, with TPO-RA-based combinations achieving response rates around 70% in heavily pretreated patients who had failed multiple prior therapies including splenectomy and rituximab [81,82,83,84].

Data on combination treatment approaches utilizing fostamatinib beyond TPO-RAs are limited but emerging. Some case reports and expert reviews suggest that fostamatinib could potentially be used in combination with immunosuppressants (such as mycophenolate mofetil or cyclosporin), IVIg, corticosteroids, and even rituximab, particularly in refractory patients [54,71,82,85]. However, similar to combinations of fostamatinib with TPO-RAs, the evidence remains largely limited to case reports and retrospective analyses, with most peer-reviewed studies emphasizing the need for prospective controlled trials to establish the safety and efficacy of fostamatinib combinations before they can be routinely recommended [54,85,86].

## 9. Future Directions

Topics and considerations for future investigation and clinical research should include an analysis of biomarkers predicting treatment response. In order to further elucidate and confirm the effectiveness of fostamatinib in the treatment of patients with ITP, the development of head-to-head trials comparing fostamatinib with TPO-RAs and rituximab should be initiated. Finally, a critical evaluation of data gaps regarding treatment discontinuation strategies should be undertaken to clarify therapeutic challenges related to transitioning patients to more effective and tolerable therapies.

## 10. Conclusions

The goals of ITP treatment are to increase platelet count and reduce the risk of bleeding. Despite significant advances, the optimal management of ITP remains a clinical challenge. Fostamatinib alone or in combination with TPO-RAs has demonstrated both efficacy and safety in various ITP patient populations [53,68,70,75]. This review provides an overview of the current ITP treatment landscape and offers guidance to clinicians for developing a personalized treatment approach that leverages clinical use of fostamatinib and TPO-RAs. This proposed treatment approach may help elucidate individual ITP pathophysiology, potentially leading to improved patient outcomes by illustrating a clear pathway for treatment adjustments based on patient response.

**Table 1:** Pharmacological therapies recommended by treatment guidelines for second-line treatment of immune thrombocytopenia.

Treatment (First FDA Approval for ITP)	Mechanism of Action	Indicated Patient Population and Initial Dose	Pivotal Clinical Trials Supporting ITP Indications	Efficacy in Second-Line or Later Settings	Safety	Drug–Drug Interactions
**Eltrombopag** [87,88,89,90,91] **(2008)**	TPO-RA that stimulates platelet production	Adults and children ≥1 year old with chronic ITP **Initial dose:** 50 mg/day (25 mg/day in patients of Asian ancestry, those with mild to severe hepatic impairment, and those age 1–5 years)	**Adult:** NCT00102739, NCT00370331 **Pediatric:** NCT00908037, NCT01520909	59% (43/73) and 70% (19/27) of adult patients experienced a platelet response ≥50 × 10^9^/L in 2 studies 60% of 134 adult patients had a sustained platelet response ^a^	Common AEs include anemia, nausea, pyrexia, ALT increased, cough, fatigue, headache, and diarrhea	Requires coordination of dose with foods or supplements that contain polyvalent cations (e.g., iron, calcium, magnesium, zinc) Use caution with coadministration of substrates of OATP1B1 or BCRP
**Romiplostim** [92,93,94,95] **(2008)**	TPO-RA that stimulates platelet production	Adults with ITP and insufficient response to corticosteroids Children ≥1 year old with ITP for ≥6 months and insufficient response to corticosteroids **Initial dose:** 1 mcg/kg	**Adult:** NCT00102336, NCT00102323 **Pediatric:** NCT01444417, NCT00515203	88% (36/41) of adult patients experienced a platelet response ≥50 × 10^9^/L 61% (25/41) adult patients had a durable platelet response ^a^	Common AEs in adults include arthralgia, dizziness, insomnia, myalgia, pain in extremity, abdominal pain, shoulder pain, dyspepsia, and paresthesia Common AEs in pediatric patients include contusion, upper respiratory tract infection, and oropharyngeal pain	None reported
**Avatrombopag** [96,97] **(2019)**	TPO-RA that stimulates platelet production	Adults with chronic ITP and insufficient response to prior treatment **Initial dose:** 20 mg/day	NCT01438840	66% (21/32) patients had a platelet response ≥50 × 10^9^/L at day 8 of treatment 32 patients had a median of 12.4 weeks with a platelet response ≥50 × 10^9^/L without rescue therapy	Common AEs include headache, fatigue, contusion, epistaxis, upper respiratory tract infection, arthralgia, gingival bleeding, petechiae, nasopharyngitis	Dose adjustments are necessary with concomitant CYP3A4 or CYP2C9 inhibitors or inducers
**Fostamatinib** [70,75,98] **(2018)**	SYK inhibitor that prevents phagocytosis of platelets by spleen macrophages	Adults with chronic ITP and insufficient response to ≥1 prior treatment **Initial dose:** 100 mg twice daily	NCT02076399, NCT02076412	44% (64/146) patients had a platelet response ≥50 × 10^9^/L 18% (27/146) responders had a stable platelet response ^b^	Common AEs include diarrhea, hypertension, nausea, respiratory infection, dizziness, ALT/AST increase, rash, abdominal pain, fatigue, chest pain, and neutropenia	CYP3A4 inhibitors may require dose modification Avoid concomitant CYP3A4 inducers
**Rituximab** [6,99,100] **(Not FDA-approved for ITP)**	Monoclonal antibody that targets CD20 on B cells to trigger apoptosis	Studies have been performed in adults and children ≥2 years old with ITP **Evaluated dose:** Commonly 4 weekly infusions of 375 mg/m^2^	–	57% (215/376) adult patients with ITP had a platelet response ≥50 × 10^9^/L 43% of 60 adult patients had a 1-year platelet response ≥50 × 10^9^/L	Common infusion-related AEs include chills, bronchospasm, neutropenia, serum sickness, increased infection risk, PML, hypogammaglobulinemia	None reported

^a^ A sustained or durable treatment response was defined as a platelet response ≥50 × 10^9^/L for 6 of the last 8 weeks of treatment without the use of rescue medication. ^b^ A stable platelet response was defined as a platelet response ≥50 × 10^9^/L for ≥4 of 6 bi-weekly visits during weeks 14 to 24 of treatment without the use of rescue medication. AE, adverse event; BCRP, breast cancer resistance protein; DVT, deep vein thrombosis; ITP, immune thrombocytopenia; OATP1B1, organic anion transporting polypeptide 1B1; PML, progressive multifocal leukoencephalopathy; SYK, spleen tyrosine kinase; TPO-RA, thrombopoietin receptor agonist.

**Table 2:** Literature on fostamatinib and TPO-RA combination treatment.

Citation	Patient Population	Efficacy	Safety	Follow-Up Period
Hughes et al. [54] (2021)	Patients transitioning from thrombopoietin agonists	Case study 1: Transitioned from romiplostim without need for rescue therapy and maintained platelet count above 100 × 10^9^/L for >1 year Case study 2: After initiating combination therapy with eltrombopag, platelet count increased to 41 × 10^9^/L and maintained for a year at 90–100 × 10^9^/L Case study 3: Transitioned to fostamatinib from romiplostim and platelet counts stabilized ≥100 × 10^9^/L Case study 4: Fostamatinib was added to rituximab and romiplostim and platelets increased to a peak of 210 × 10^9^/L and patient has maintained normal platelets Case study 5: Fostamatinib was added to avatrombopag, and platelet count rose to 366 × 10^9^/L and stabilized	No AEs reported	Patient-dependent; ranged from 2 months up to 22 months at the time of publication
Passucci et al. [53] (2024)	Relapsed or refractory chronic ITP patients who received fostamatinib with prednisone or TPO-RAs as a bridge to fostamatinib monotherapy or time-limited continuous treatment (N = 15)	Twelve of fifteen (80%) achieved a response, with a median time to response of 9 days 73% (11/15) achieved a complete response, with a median time to best response of 13 days	New-onset arterial hypertension: 20% (3/15) Diarrhea: 20% (3/15) No DVT, severe AEs, or extreme thrombocytosis occurred Treatment discontinuation: 20% (3/15) due to gastrointestinal symptom grade <3 or ITP relapse	Median (IQR), 119 days (38–245)
Mingot-Castellano et al. [55] (2024)	Patients with multirefractory ITP (N = 18)	Combination response achieved in 83.3% of patients with a median time from treatment initiation to best response of 15 days	Treatment-related AEs: 33% (6/18) Most frequently occurring: gastrointestinal (15%), vascular events (13%), and infections (13%) No deaths occurred One discontinuation: asymptomatic neutropenia grade 3	Median (IQR), 256 days (142.8–319)

AE, adverse event; DVT, deep vein thrombosis; ITP, immune thrombocytopenia; TPO-RA, thrombopoietin receptor agonist.

## Data Availability

Data sharing is not applicable to this article as no datasets were generated or analyzed during the current study.
